# Supplementary material for: Development of a specific troponin I detection system with enhanced immune sensitivity using a single monoclonal antibody
Source: R Soc Open Sci. 2020 Oct 7;7(10):200871. doi: 10.1098/rsos.200871 (PMC7657922; doi:10.1098/rsos.200871)
Supplement: Supplementary Information [file rsos200871supp1.pdf]

ROYAL SOCIETY
OPEN SCIENCE

R. Soc. open sci.
doi:10.1098/not yet assigned

Development of a Specific Troponin I Detection System with Enhanced Immune Sensitivity using a Single Monoclonal Antibody

Anıl Bozdogan [1,2], Reham F. El-Kased [3]*, Vanessa Jungbluth [2], Wolfgang Knoll [1,2], Jakub Dostalek [2]*, Amal Kasry [1,4]*

1 CEST Competence Centre for Electrochemical Surface Technology, 2700 Wiener Neustadt, Austria.
2 Biosensor Technologies, AIT-Austrian Institute of Technology GmbH, Konrad-Lorenz-Straße 24, 3430 Tulln, Austria.
3 Department of Microbiology and Immunology, Faculty of Pharmacy, The British University in Egypt (BUE), El-Sherouk City, Suez Desert Road, Cairo 11837, Egypt.
4 Nanotechnology Research Centre (NTRC), The British University in Egypt (BUE), El-Sherouk City, Suez Desert Road, Cairo 11837, Egypt

Supplementary Information

Raw data of Figures 2, 3, and 4: are submitted as electronic supplementary materials.

Blast results of cTnT and cTnI:

The epitope (87 – 91); GLGFA is not found through the whole Troponin T sequence.

cTnT:

```
        1 msdieevvee yeeeeqeeaa veeqeeaaee daeaeaetee traeedeeee eakeaedgpm
       61 eeskpkprsf mpnlvppkip dgervdfddi hrkrmekdln elqalieahf enrkkeeeel
      121 vslkdrierr raeraeqqri rnerekerqn rlaeerarre eeenrrkaed earkkkalsn
      181 mmhfggyiqk aqterksgkr qterekkkki laerrkvlai dhlnedqlre kakelwqsiy
      241 nleaekfdlq ekfkqqkyei nvlrnrindn qkvsktrgka kvtgrwk
```

cTnI:

```
        1 madgssdaar eprpapapir rrssnyraya tephakkksk isasrklqlk tlllqiakqe
       61 lereaeerrg ekgralstrc qplelaglgf aelqdlcrql harvdkvdee rydieakvtk
      121 niteiadltq kifdlrgkfk rptlrrvris adammqallg arakesldlr ahlkqvkked
      181 tekenrevgd wrknidalsg megrkkkfes
```
